# Reconstruction of malunited diaphyseal fractures of the forearm

**DOI:** 10.1007/s11552-014-9635-9

**Published:** 2014-05-01

**Authors:** Prakash Jayakumar, Jesse B Jupiter

**Affiliations:** The Hand and Upper Extremity Service, Massachusetts General Hospital and Harvard Medical School, 55, Fruit Street, Boston, MA 02114 USA

**Keywords:** Forearm, Fracture, Malunion, Malunited, Diaphysis, Diaphyseal, Reconstruction, Deformity, Correction, Osteotomy

## Abstract

The forearm is a complex anatomical and functional unit with unique osseous, soft tissue and articular relationships. Disruption of these important relations can have a significant impact, leading to pain, instability of the radio-ulnar articulation and reduced range of motion. The gold standard for treating forearm fractures in adults remains anatomic reduction, stable plate fixation and preservation of the surrounding blood supply. Failure to achieve these goals may lead to malunion, requiring reconstructive surgery, which can be technically challenging. In this review, we discuss the essential aspects of anatomy and pathomechanics, clinical and radiological assessment and the state of the art in pre-operative planning and deformity correction surgery.

## Background

Malunited fractures of the forearm can be difficult problems, and reconstructive surgery can be technically challenging. This is a direct consequence of the complex interplay between the bone and soft tissue elements of the forearm that facilitate the positioning and orientation of the wrist and hand in space. Moreover, the forearm is the principal contributor to rotation of the upper limb when the shoulder is fully abducted [25]. Thus, malunion can lead to reduced forearm rotation, pain and instability of the distal radio-ulnar joint (DRUJ) [36, 54].

The gold standard for treatment of forearm fractures in adults involves anatomic reduction, stable plate fixation and preservation (and optimisation) of biology as established by the Arbeitsgemeinschaft für Osteosynthesefragen (AO) group [34]. Adhering to these principles minimises both non-union and malunion while achieving good to excellent functional outcomes [1, 7, 10, 25, 36, 43]. In contrast, non-operative treatment using closed reduction and cast immobilisation provides limited control and stability in these fractures [25].

Restoring structure and function of the malunited forearm requires a thorough understanding of the normal as well as pathological anatomy, combined with a thorough clinical assessment and acquisition of appropriate imaging. We discuss these essential aspects alongside the state of the art and future directions in pre-operative planning and principles of deformity correction.

## Anatomy and Pathomechanics of Malunion

The forearm should be conceptualised as a single bicondylar articulation [25]. Optimal treatment of acute and malunited forearm fractures is aligned with the AO principles of restoration of anatomy, stable fracture fixation and preservation of blood supply with early mobilisation, taking into account bony, soft tissue and joint-related factors [34].

The radius and ulna form a dynamic functional unit with unique proximal and distal articulations. The radius has a physiological bow and rotates around a stationary ulna during pronation and supination. The longitudinal axis of the forearm bisects the centre of the radial head and the distal ulnar fovea.

The interosseous membrane (IOM) spans the length of the forearm, contributing to its longitudinal stability [22, 38, 41]. The central and dorsal oblique bands provide axial and proximal radio-ulnar joint (PRUJ) stability, respectively. Secondary DRUJ stability is provided by the distal membranous portion [38, 52]. The supinator, pronator teres and pronator quadratus muscles exert deforming forces upon fracture fragments leading to narrowing of the interosseous space and altered rotation [57]. The supinator, pronator teres and pronator quadratus insert into the proximal, middle and distal thirds of the radius, respectively. Diaphyseal fractures occurring proximal to the pronator teres insertion will result in the proximal fragment being supinated and flexed by unopposed action of the supinator and biceps brachii, while the distal fragment will pronate due to the force of the pronator quadratus and pronator teres. Fractures distal to the pronator teres insertion will result in the proximal fragment maintaining neutral rotation as the supinator counteracts the the action of the pronator teres and flexion due to the action of the biceps brachii. The distal fragment is pronated and deviated toward the ulna by the pronator quadratus. Flexor muscles of the forearm tend to displace the distal fragments of the forearm anteriorly leading to dorsal bowing of the radius and ulna. These muscle-deforming forces may lead to dorso-volar or radio-ulnar angular, axial or combined deformities. Studies demonstrate residual motion may be impeded despite anatomic fracture reduction due to soft tissue contracture [53].

### Joint-Related Factors

The PRUJ and DRUJ are the only points of contact between the radius and ulna. The radio-ulnar articulation is stabilised proximally by the elbow joint capsule and annular ligament and distally by the triangular fibrocartilage complex (TFCC).

The pathomechanics of forearm malunion is directly related to disruption of the radio-ulnar relationship, leading to altered motion and potential instability. Morrey et al. recognised that performance of most activities of daily life requires 50° each of pronation and supination [33]. A higher range of forearm rotation may be required for modern activities [46]. The rotational arc is variably impeded by dorso-volar and radio-ulnar angular, axial or combined deformities.

*Angular deformities* of the radius and ulna increase IOM tension leading to bony impingement and restricted radial rotation about the mechanical axis [19]. *Axial rotational deformities* also lead to stiffness and restriction of forearm rotation due to malalignment and abnormalities in the radio-ulnar articulation [11, 19, 26, 53, 55]. A variety of biomechanical and cadaveric studies demonstrate these effects [9, 11, 26, 29, 36, 40, 47, 53, 55]. Key factors associated with angular deformity include the *degree of angulation*, *location* of deformity and one or both *forearm bone involvement*.

### Degree of Angulation and Location of Deformity

Studies consistently demonstrate that clinically significant limitations of forearm rotation occur after 15–20° of radio-ulnar or dorso-volar angular deformity [29, 47, 53]. Matthews et al. demonstrated that diaphyseal angulations of 10° or less in the dorso-volar direction or toward the IOM did not alter forearm motion. However, angulations of 20° in the radius or ulna in either direction caused 30 % loss of pronation–supination and functionally important restrictions to rotation [29]. A cadaveric study by Tarr et al. also demonstrated that combined total angular deformities (radio-ulnar or dorso-volar) of 10° or less resulted in losses of 18° or less, while 15° of total deformity resulted in loss of motion greater than 27° [53]. While the loss in range of pronation was similar in both middle and distal forearm deformities, the loss of supination was only minimal for the distal third but severe in the middle third. Anatomically, the middle third is the zone where both forearm bones overlap at extreme pronation and supination. Furthermore, the central three fifths of the radius form the major lateral convexity of the radius [53]. Proximal deformities have less impact on the range of motion than those deformities of similar magnitude in the distal two thirds of the radius [47].

### Forearm Bone Involvement

Matthews et al. demonstrated that 10° of angulation in the dorso-volar or radio-ulnar direction of one forearm bone had minimal impact on range of motion, while combined deformities of 10° involving the radius *and* ulna toward the IOM resulted in a significant reduction in supination [29]. Isolated angulations of the radius in the order of 20° demonstrated reduced pronation with dorsal angulation, supination with volar angulation and both supination and pronation with angulation toward the IOM [53]. Combined angular deformities of both bones in different directions significantly restrict range of motion compared with both bone deformities in the same direction [18, 47, 53]. Effects of axial malunions are determined by degree of rotational deformity and which forearm bones are involved [11-13, 26, 36, 53, 55].

### Degree of Rotation

Pure rotational deformities produce losses in pronation–supination equal to the degree of deformity [14]. Physiological limits of individual variations in forearm rotation range up to 30° for the radius and 20° for the ulna. Although accurate clinical assessment of malunions within this range may be difficult [11-13, 29, 36, 54], they provide useful thresholds for treatment with deformity correction surgery [6].

### Forearm Bone Involvement

Dumont et al. investigated isolated and combined axial rotational malunions of the radius and ulna [11]. Isolated rotational malunion of the radius in supination causes a significant reduction in forearm rotation especially if the malunion is greater than 60° [11, 36]. Malunion of the radius in pronation demonstrates a corresponding limitation in supination [36]. In contrast, isolated rotational malunion of the ulna in supination is shown to have minimal effects on forearm rotation [11]. Pronatory ulna malunions only moderately reduce forearm rotation and decrease supination to a lesser extent compared with pronatory malunions of the radius [11, 36]. A cadaveric study by Tynan et al. simulating ulnar rotational malunion demonstrated a decrease in forearm rotation in one direction while increasing range of motion in the opposite direction, thus maintaining the total arc of forearm rotation [55]. Rotational deformities of both bones in the same direction had a similar effect to isolated malunions of the radius, while combined deformities in opposite directions resulted in the most significant restriction to both pronation and supination [11, 36].

Combined axial and dorso-volar or radio-ulnar angular deformities not surprisingly cause marked restriction in range of motion and may be associated with DRUJ dysfunction [11, 36, 52]. DRUJ instability and pain may occur following these deformities or length discrepancies of the radius and/or ulna [36, 52]. Disruption of the soft tissue constraints of the DRUJ may lead to instability, subluxation, dislocation and an incompetent TFCC [5, 52]. Apex volar angulation and axial malunion of the radius in pronation in the context of Galeazzi fractures is associated with dorsal ulnar subluxation and complete loss of active supination [25]. Conversely, apex dorsal angulation leads to volar subluxation of the distal ulna.

PRUJ disruptions most commonly involve a chronically dislocated radial head following forearm malunion and radio-ulnar length discrepancy, as well as a persistently angulated ulna associated with Monteggia fractures. The former occurs following neglected subluxations in high-energy trauma or injuries involving concurrent proximal and distal forearm fracture–dislocations [15, 20]. Persistent dislocation often results from inadequate fracture reduction leading to relative ulnar shortening, rather than disruption of the proximal IOM and ligamentous restraints of the radio-capitellar joint. Proximal radial malalignment may lead to severe rotational limitations due to radial head dislocation.

## Clinical Presentation

Forearm malunions may occur following either non-operative or operative treatment of acute fractures as well as following deformity correction surgery.

### Clinical Assessment

Clinical history may elicit pain, stiffness, loss of motion, loss of power and disability. Painful forearm rotation may be related to bony impingement and tensioning of the IOM secondary to angular deformity. It may also occur due to abnormal joint kinematics following radio-ulnar joint malalignment secondary to axial malunion. Clinical examination should define the restriction in range of forearm motion, particularly pronation and supination, and ascertain signs of DRUJ instability [54].

#### Imaging

Radiographic assessment includes postero-anterior (PA) and lateral views of the forearm in neutral rotation plus maximal pronation and supination. PA radiographs can be obtained with the arm placed on the imaging plate with the shoulder at 90° of abduction and elbow at 90° of flexion. The beam is orthogonally directed toward the forearm in neutral position in the PA direction. Contralateral radiographs should be acquired for pre-operative templating although important side-to-side differences have been demonstrated in healthy populations [1, 14]. Wrist and elbow radiographs, including the DRUJ and PRUJ, should be acquired with antero-posterior (AP), lateral and two oblique views.

**Fig. 1:**
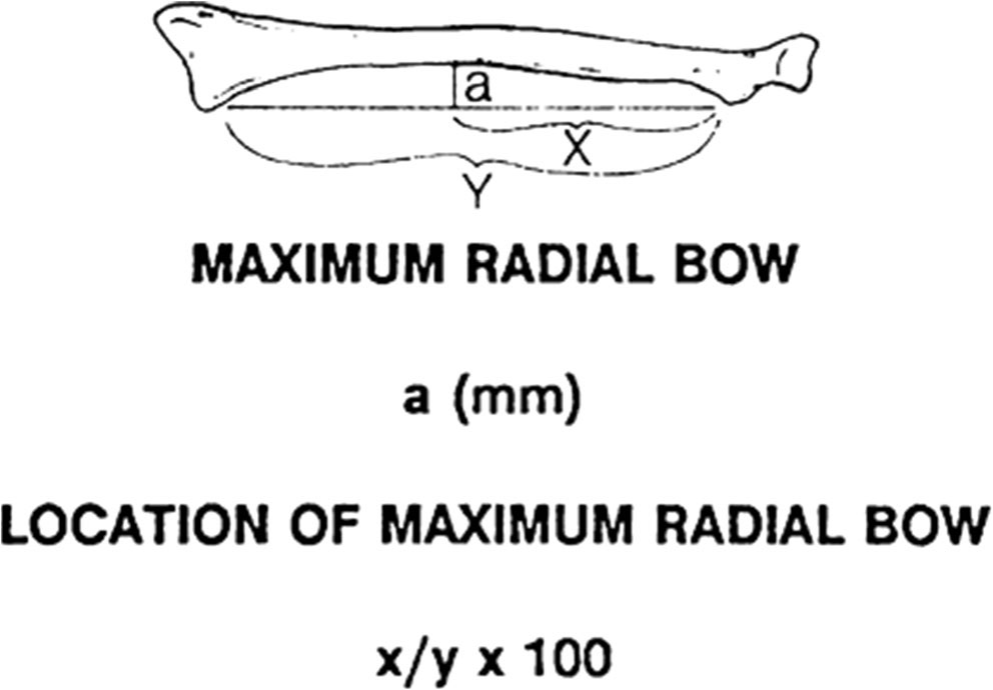
Measurement of the magnitude and location of the maximal radial bow. Using an AP forearm radiograph in neutral, including the wrist and elbow, a line is drawn from the midpoint of the bicipital tuberosity to the ulnar-most aspect of the distal radius to form a baseline (*y*, mm). A *perpendicular line* is drawn from the baseline to the point of maximal radial bow (*a*, mm) and represents the magnitude. The distance from the bicipital tuberosity to the intersection of this perpendicular line (*x*, mm) represents the location as a percentage proportion of the baseline (*x*/*y* × 100). (Adapted from Schemitsch and Richards [48]; permission acquired)

The *relative length* of the radius and ulna should be assessed, including degree of ulnar variance, in comparison with the contralateral side, providing an indication of length restoration required. Schemitsch et al. define the magnitude and location of maximal *radial bow* based on contralateral radiographs [48] (Fig. 1). *Angular* deformities are assessed on true AP and lateral views. *Rotational* deformities are more difficult to assess and require assessment of the relationship between the radial styloid and bicipital tuberosity and ulnar styloid and coronoid process on full forearm views comparing both sides [2, 12]. The level of axial malunion may be defined by comparison with the level of the original fracture or the level of angular deformity in patients with combined deformity.

Comparative CT or MRI may assess rotational deformity more effectively using cross-sectional images in pronation and supination [2, 12, 13, 36]. Proximal and distal axial slices of both forearms are compared to quantify the degree of rotation using bony edge reference points at the bicipital tuberosity and level of Lister’s tubercle, and ulnar styloid and trochlea, for radial and ulnar torsion, respectively [2, 12, 44]. A standardised, reliable and reproducible method for assessing forearm rotation is still required [11-13].

### Pre-operative Planning

Pre-operative planning is essential in deformity correction surgery for malunited forearm fractures [4]. Early experience of computer-aided planning has been promising [17, 24, 28, 50]. It has been utilised for simulating pre- and post-operative motion and integrated into software for planning osteotomies in complex diaphyseal malunions with good functional results [17, 50].

**Fig. 2:**
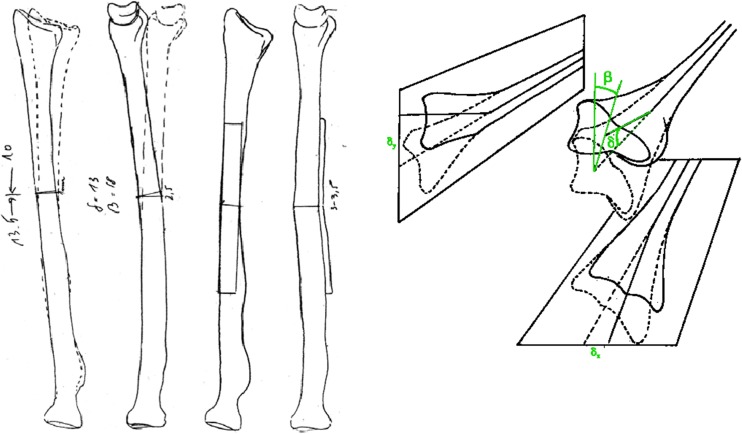
A step-by-step guide to deformity correction. (1) Superimpose the contours of the forearm bone in malunited position with the anatomical position. (2) Define the maximum point of deformity and determine the osteotomy site. (3) Measure the angles of correction required, calculating *α*, *β* and *δ* angles. (4) Check the angles against standardised reference tables. (5) Draw the osteotomy wedge and measure the wedge height. (6) Prepare the plate and measure the plate offset..(7) Close the osteotomy and stabilise

**Fig. 3:**
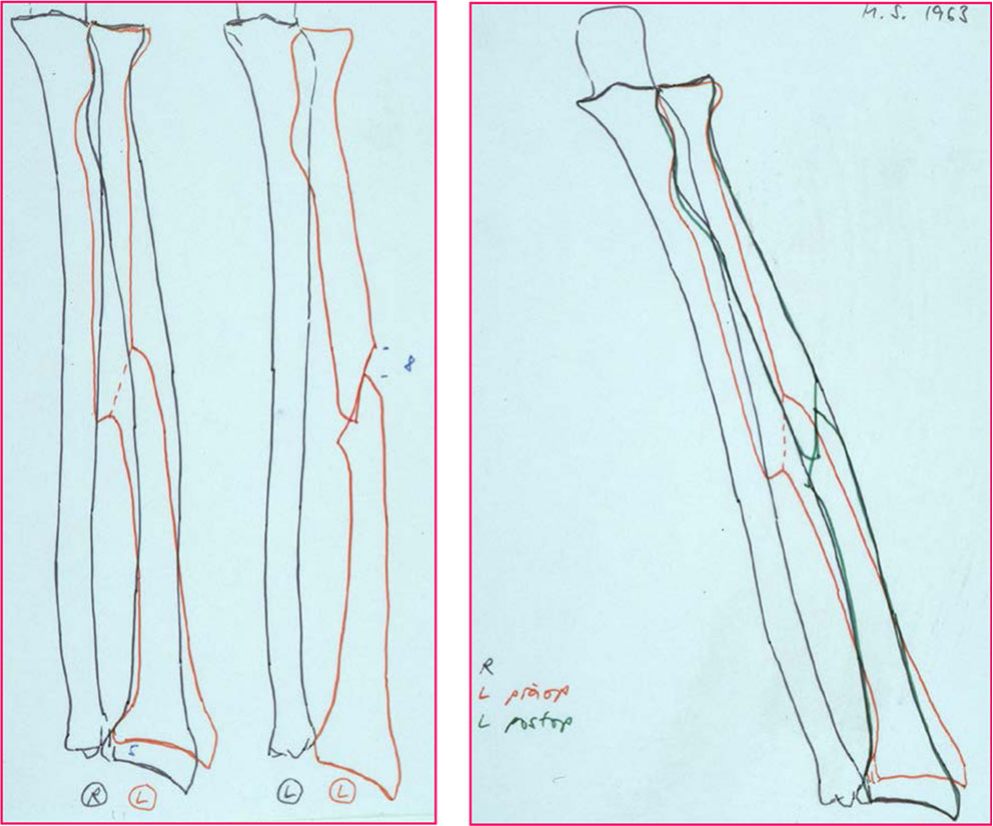
The concept of overlay drafting. The contours of the major fracture fragments of the malunited forearm are superimposed over the outline of the “normal” contralateral side, using tracing paper, cutouts or electronic tablet devices linked to a computer. (Images courtesy of D. Fernandez [25]; permission acquired)

**Fig. 4:**
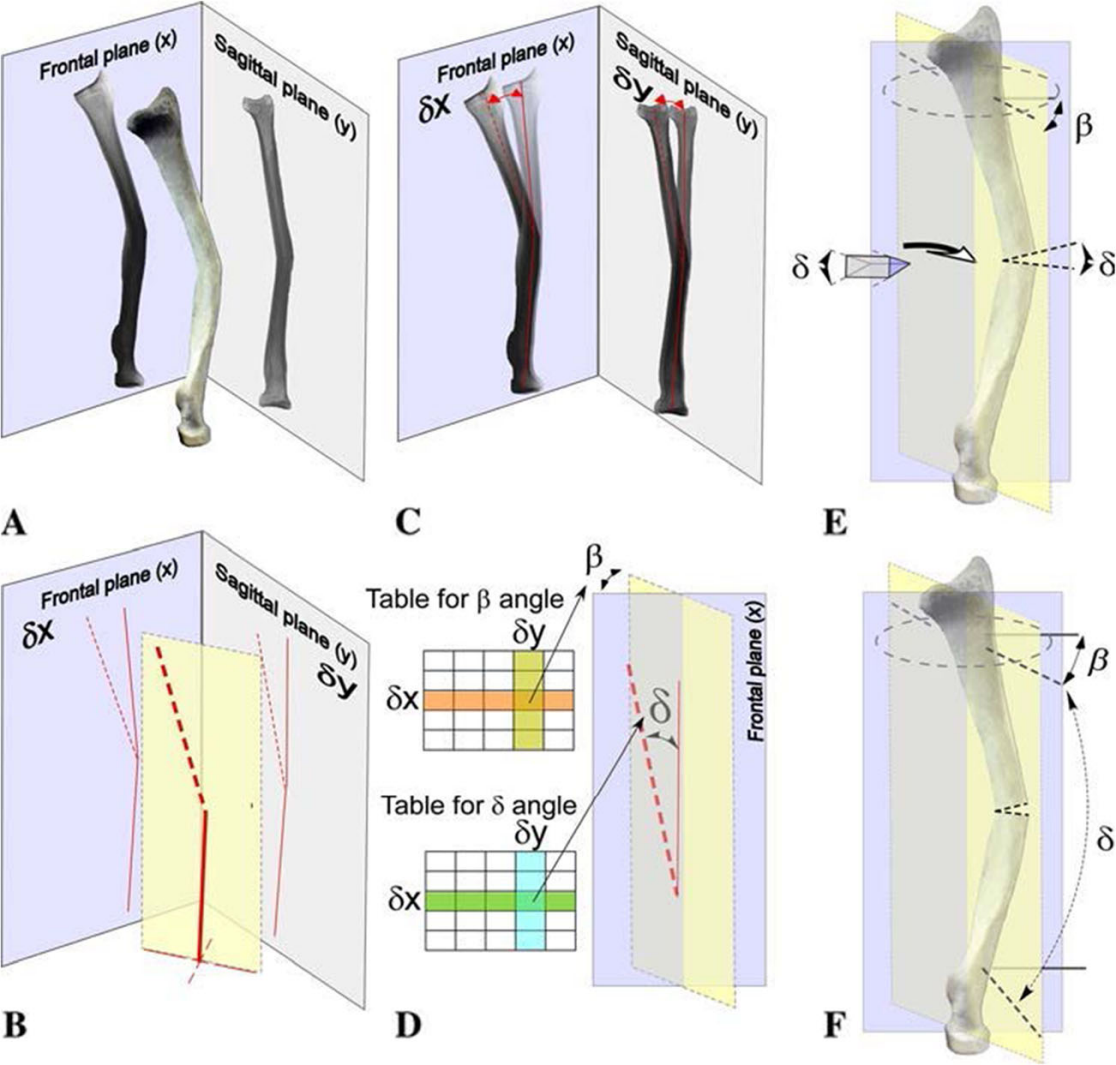
Malunion involving radio-dorsal and ulnar-volar angular deformity of the middle third of the radius. **a** Orthogonal radiographs are used to assess the orientation and value of the maximal angular deformity, and **b** projections are measured in the frontal (*δx*) and sagittal planes (*δy*). **c** Pre-operative planning starts with superimposition of radiographs from both sides allowing angular deformity assessment in both planes. **d** Established tables [37] factor in *δx* and *δy* to assess the true angle of deformity (*δ*) and orientation of deformity in space (*β*). **e** *δ* defines the angle required for a closing wedge osteotomy or structural bone graft for open wedge osteotomy depending on correction required. **f** Correction is performed in the plane of maximum deformity, i.e. *β* in relation to the frontal plane. Intra-operatively, two Kirschner wires (K-wire) (*plain line*) are placed in the frontal plane using the distal radius as a landmark. The osteotomy level also is marked with a K-wire. Finally, the plane of correction is marked with two K-wires (*dotted line*) inserted with a *β* angle with respect to K-wires in the frontal plane. The second of these wires is inserted with a *δ* angle in relation to the first. Both K-wires should be parallel after osteotomy, i.e. *δ* = 0 and *β* angle stays the same. (Adapted from L. Nagy, L. Jankauskas and C.E. Dumont [36]; permission acquired)

A stepwise approach is essential (Fig. 2). The concept of overlay drafting using the contralateral forearm as a reference can be used to calculate the degree of correction required (Fig. 3). Nagy et al. utilise these principles in quantifying the *orientation of deformity in space* by defining the *true angle of deformity* in *angular* malalignments [36] (Fig. 4). The point of maximal deformity represents the osteotomy site, the plane of maximal deformity represents the direction for the correction and the true angle determines the wedge angle that should be removed for a closing wedge correction or the angle that should be factored into a structural bone graft for open wedge correction. Once the maximal point is defined, corrective opening or closing wedge osteotomy can be performed based on the type of malunion and desired correction.

### Treatment

#### Operative Rationale

The basis of forearm malunion surgery is consistent with the fixation of forearm fractures in general, namely the restoration of length, angular and rotational alignment, and displacement as well as the radial bow [48]. Operative indications include intractable pain, deformity, radio-ulnar joint instability, functional limitations and restricted forearm range of motion. Forearm rotation is significantly limited with dorso-volar or radio-ulnar angular deformity greater than 15°, radial malrotation greater than 30° and ulnar malrotation greater than 20° compared to the contralateral side [11, 29, 46, 53]. The goal of operative intervention is to achieve 50° of pronation and 50° of supination [33].

#### Operative Technique

Surgical exposure for forearm malunion commonly utilises the Henry approach and direct ulnar approach [21]. The radial diaphysis is fully accessed through the release of the pronator teres. Proximal extension allows access to the PRUJ and proximal radius with release of the supinator while protecting the posterior interosseous nerve. The ulna is approached through the interval between the flexor and extensor carpi ulnaris. The approach is fashioned according to the type of osteotomy, which itself is dependent on the type of malunion and correction required [21].

*Transverse* osteotomies are conducted for “simple”, isolated rotational or translational deformities where no angulatory correction is required [25]. The point of maximal rotation defines the osteotomy site where the bone is cut in a transverse plane. De-rotation is performed using wire markers.

**Fig. 5:**
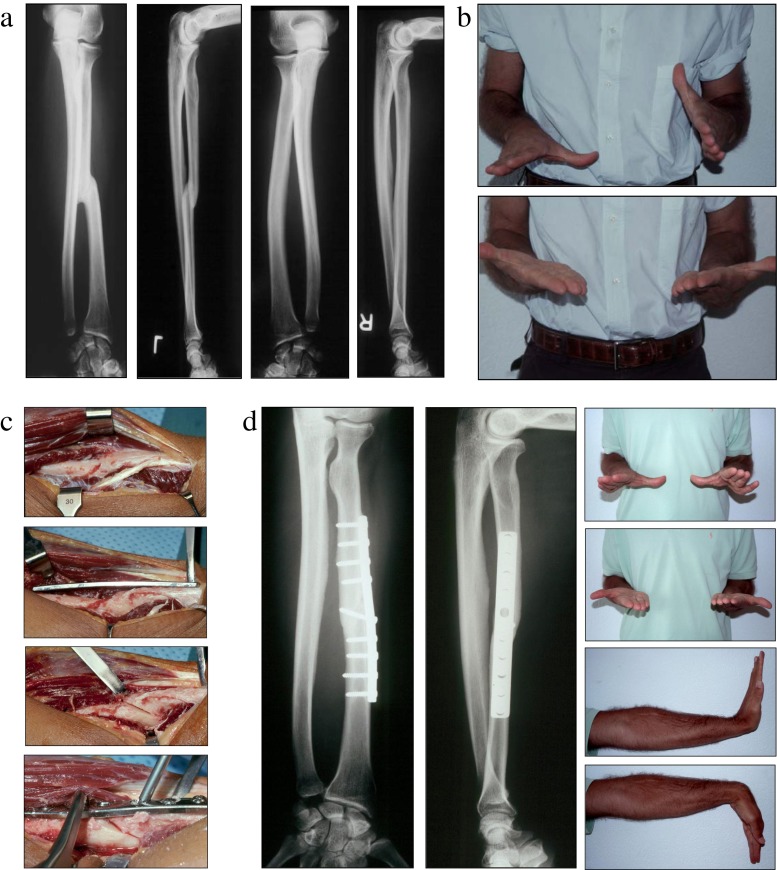
Oblique corrective osteotomy for a malunited diaphyseal fracture of the forearm. **a** Comparison radiographs—AP and lateral views of the left forearm malunion and the normal contralateral side. **b** Clinical demonstration of pre-operative rotational profile. **c** Operative technique with exposure of the malunion site, plate contouring, oblique osteotomy and stabilisation of the osteotomy site. **d** Post-operative radiographs—AP and lateral views and post-operative range of motion

*Oblique* osteotomies are conducted for angular corrections in the plane of the deformity with moderate lengthening [30, 44] (Fig. 5). These are not recommended for rotational correction as rotation at the site may translate to excessive angular malalignment, opening the osteotomy and reducing the contact surface area.

**Fig. 6:**
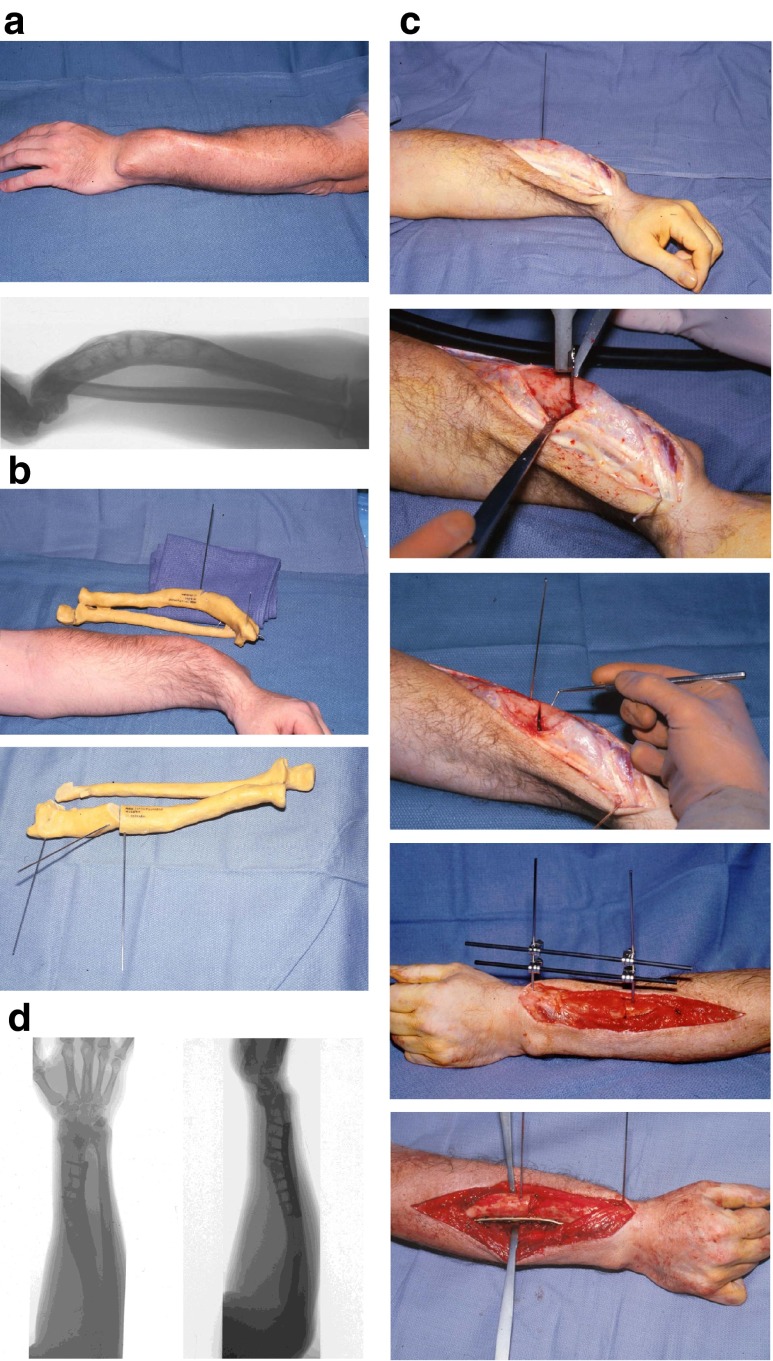
Single-cut corrective osteotomy for a malunited diaphyseal fracture of the forearm. **a** Pre-operative clinical image and radiograph of a complex malunion of the forearm. **b** CT-guided development of a plastic model of deformity to aid pre-operative surgical planning and simulation of bone wedge excision in the true plane of deformity. **c** Operative technique with exposure of the malunion site, single-cut osteotomy, temporary stabilisation with external fixator and definitive plate stabilisation of the osteotomy site. **d** Post-operative radiographs—AP and lateral views

“*Single-cut”* osteotomies are conducted for complex combined deformities requiring angular, axial and length corrections (Fig. 6). The osteotomy is orientated in the combined oblique plane of deformity, addressing both malrotation and angular malalignment in two planes. Computerised mathematical analysis is often utilised to calculate corrections [31, 45]. Residual length discrepancy may require an opening or closing wedge osteotomy in combination with the single-cut osteotomy. Osteotomies may allow lengthening to address these discrepancies with or without the requirement of additional bone grafting. Distraction osteogenesis should be utilised for lengthening of more than 4 cm. Extreme radial bowing or segmental malunions may require double-level osteotomies [31, 45].

The general technique for performing osteotomies involves marking the site of maximal deformity (apex), the level of osteotomy and planes of correction in both fragments with Kirschner wires under fluoroscopy (Fig. 4). These are usually based on pre-operative radiographic measurements. Limited-contact compression plates (LC-DCP), 3.5 mm, are contoured and fixed temporarily to the proximal fragment. The plate is then removed, osteotomy is performed and the wedge excised. Reduction is achieved and checked under fluoroscopy with orthogonal views prior to definitive fixation. Single-screw fixation is achieved, and passive rotation is reassessed manually and radiographically. The IOM should be assessed before and after osteotomy. Contractures may limit functional gains despite the restoration of bony anatomy. Although IOM release or partial resection for post-traumatic stiffness secondary to malunion is shown to allow further correction, it is rarely performed [29, 36, 47, 54, 58]. It is unclear whether it significantly improves forearm rotation or conversely restricts range and causes DRUJ instability, weakness and radio-ulnar synostosis. Ultimately, angular or translational deformities reduce interosseous space and lead to IOM contracture. This may be precluded by early surgical intervention [19].

External fixator application may also be required in severe deformities with significant soft tissue contracture. This may optimise soft tissue tension prior to definitive fixation [25].

In both bone malunions, some authors recommend that the bone with greater deformity should be corrected first [54] while others suggest initial realignment of the ulna [25]. In the former, stabilisation of the initial bone may be sufficient in restoring range of motion precluding the requirement for an osteotomy of the second bone. In the latter, initial ulnar correction allows further realignment of the radius, correcting length and angular malalignment and achieving more precise congruity of the radio-ulnar joints.

### Post-operative Management

Early passive exercises should progress to active assisted range of motion exercises at 1 to 2 weeks following surgery. Strengthening exercises should commence at 6 to 8 weeks. Full weight-bearing and contact sports should be allowed once union is confirmed.

### Complications

Complications include non-union, delayed union, refracture, heterotopic ossification, radio-ulnar synostosis, radio-ulnar joint instability and residual loss of motion. Heterotopic ossification and radio-ulnar synostosis may occur following contact with the morselised bone graft used for augmentation. Radio-ulnar instability, subluxation and residual loss of motion can occur with or without pain [57]. A painful, unstable DRUJ may require imaging or arthroscopic evaluation of the TFCC [51, 52]. Complex deformity correction is fraught with a high risk of technical error resulting in residual loss of motion. Loss of rotation, in general following forearm malunion surgery, requires thorough pre-operative counselling.

## Discussion

Reconstructive surgery to restore the anatomy and function of malunited forearm fractures demands thorough pre-operative planning and meticulous technique to achieve deformity correction, stable fixation, solid union and pain-free motion [35, 50]. Particularly important anatomical features to consider include the radial bow, proximal and distal radio-ulnar articulations and IOM [19, 48].

Schemitsch et al. investigated 55 patients with both bone forearm fractures treated with open reduction internal fixation [48]. Reconstruction of the maximum radial bow by restoring its magnitude and location along the radius had a significant impact on functional outcome. Restoring the location of the maximal bow to within 4 % of the contralateral side achieved a minimum of 80 % of normal rotation and to within 5 % of the contralateral side achieved a minimum of 80 % of normal grip strength. Restoring the amount of maximal bow to within 1.5 mm of the contralateral side achieved a minimum of 80 % of normal rotation. These findings have not always been reproducible [18]. Moreover, although restoration of bony anatomy is key, early range of motion during rehabilitation and soft tissue management are equally important.

Outcomes assessment in forearm fracture surgery in general demonstrates good overall function based on patient-focused health-related questionnaires, such as the disability of the arm, shoulder and hand (DASH) score, without corresponding improvements in clinimetric measures such as range of motion, grip strength and radiographic parameters [18]. This highlights the potential disparity between subjective and objective outcomes assessment in these injuries and the ability of the former to more accurately assess the health status.

Timing of surgery is also important, both in relation to skeletal maturity and duration since the initial fracture. Corrective osteotomies performed in children under 10 years demonstrate greater increases in range of motion than those in older children, likely due to increased growth potential [8, 16, 23, 39, 56]. Early performance of corrective surgery is also recommended [54, 56]. Trousdale et al. investigated 27 consecutive osteotomies for forearm malunions and demonstrated improved range of motion and fewer complications in cases conducted within 12 months following the initial injury [54].

Multiplanar corrective osteotomies for reconstructing complex forearm malunions are technically challenging. Computerised three-dimensional geometric modelling has been utilised to dynamically simulate the functional effects of forearm malunion as well as augment pre-operative planning for complex corrections [3, 31, 35, 45, 57].

Three-dimensional geometric models of forearm bones and IOM have been developed to simulate the limitations in pronation and supination associated with frontal and sagittal plane angular deformities and narrowing of the IOM [57]. Significant loss of pronation–supination was observed, associated with narrowing of the IOM when the normal axis deviated more than 2 cm radio-ulnarly and 0.8 cm antero-posteriorly. Models also demonstrated the magnitude and direction of angular deformity leading to this loss, i.e. 14° radial, 7° ulnar, 5° anterior and 4° posterior.

Computer-aided pre-operative planning studies for combined angular and axial deformities with pro-supination deficits have also been conducted [27, 32, 35, 49]. This is particularly useful in assessing correction of rotational deformities, which can be difficult based on radiographs alone. This allows virtual planning of osteotomies by matching three-dimensional models of the malunited side to the “normal” side [32, 35, 54]. The process involves the definition of both proximal radial segments and quantifies the degree of angular and rotational deformity based on the deviation of distal segments [32]. Processing time is relatively quick, and early results of osteotomies performed for diaphyseal forearm malunions using computer guidance demonstrate good functional outcomes and improved resultant arcs of rotation [27, 28, 32, 35].

This technology has been utilised to manufacture actual-sized plastic bone models that aid planning and intra-operative contouring of the plate, as well as customised patient-specific osteotomy cutting guides [27, 28, 32, 33]. Forearm to forearm variability in the normal population is well documented, and computerised simulations using mathematical models are being developed to avoid imaging the opposite forearm as well as incorporating the biomechanical effects of the IOM [11-13, 53]. Further work is required to enable the use of this technology in regular practice.

Malunited diaphyseal fractures of the forearm require thorough clinical, radiographic and pre-operative assessment with an early decision for corrective deformity surgery. Both anatomical restoration and early post-operative rehabilitation are required to achieve optimal functional outcome in this technically challenging problem. Complication rates are generally higher with reconstructive surgery for malunions, and pre-operative patient counselling, education and management of expectations are essential [42].
